# Healthy Food Corners as Spatial Nudges: Consumer Awareness, Perceived Necessity, and Design Preferences in Convenience Stores

**DOI:** 10.3390/nu18182971

**Published:** 2026-09-10

**Authors:** Jaewon Lee, Hae Jin Park, Suah Moon, Gaeun Yeo, Jieun Oh

**Affiliations:** 1 Department of Nutritional Science and Food Management, Ewha Womans University, 52, Ewhayeodae-gil, Seodaemun-gu, Seoul 03760, Republic of Korea; jjenny714@ewhain.net (J.L.); jin96@ewhain.net (H.J.P.); msa2926@ewhain.net (S.M.); 2 College of Science and Industry Convergence, Ewha Womans University, 52, Ewhayeodae-gil, Seodaemun-gu, Seoul 03760, Republic of Korea

**Keywords:** healthy food corner, convenience stores, spatial nudge, S-O-R framework, perceived necessity, visual merchandising

## Abstract

Background/Objectives: Healthy Food Corners (HFCs) are zones in Korean convenience stores meant to promote healthier choices, yet how consumers respond to them remains unclear. Drawing on the Stimulus–Organism–Response (S-O-R) framework, this study examined consumer awareness and the perceived necessity of HFCs, identified preferences for their spatial and visual design, and exploratorily tested whether perceived necessity links awareness to healthy-food purchase intention. Methods: A cross-sectional online survey of 409 convenience store users aged 14–59 years in the Seoul metropolitan area was analyzed using hierarchical regression and bootstrapping mediation. Results: A descriptive contrast was observed between awareness and perceived necessity: awareness of the HFC was low (three-item composite M = 2.90 ± 1.66), whereas perceived necessity was substantially higher (M = 4.86 ± 1.25), indicating that consumers valued the concept even with limited prior exposure. Consumers preferred HFCs in ready-to-eat refrigerator sections, with clear zoning labels, horizontal grouping, green color schemes, and eye-level placement, and these preferences differed by age. In an exploratory mediation analysis, awareness predicted perceived necessity, which in turn strongly predicted purchase intention; the indirect effect was significant (0.188, 95% CI [0.128, 0.251]) while the direct effect was non-significant, a pattern consistent with full mediation. Because perceived necessity was assessed with a single item, this pathway is interpreted cautiously. Purchase intention was self-reported rather than behaviorally assessed. Conclusions: Overall, the findings suggest that HFCs may function as distinct, strategically positioned spatial nudges; however, design recommendations are based on consumer-stated preferences and require confirmation through behavioral research. The results have implications for food environment policy in Korea, with the caveat that generalizability to other countries and retail systems warrants caution.

## 1. Introduction

Promoting access to healthy foods within everyday retail environments has emerged as a central public health priority across many countries [1]. Diet-related noncommunicable diseases—including obesity, type 2 diabetes, and cardiovascular disease—continue to burden health systems worldwide, and the food environments in which people make daily choices are increasingly recognized as modifiable determinants of dietary behavior [1]. In this context, small-format retail outlets such as convenience stores have attracted growing policy attention, given their high accessibility, extended operating hours, and proximity to residential and school areas—conditions that make them particularly influential in shaping routine food choices, especially among young consumers [2,3]. Audits of convenience store assortments, however, indicate that nutrient-dense choices remain scarce within these outlets: across six stores in the United States, fewer than 9% of items containing fruits or vegetables were classified as products to be selected often, and only a single item supplying a full serving of fruits or vegetables met that standard [4]. Availability alone therefore does not guarantee healthier selection, suggesting that how healthy options are organized and made salient within the store may matter as much as whether they are stocked at all.

In Korea, the Ministry of Food and Drug Safety (MFDS) launched the “Healthy Food Corner” (HFC) pilot project in 2022, designating specific zones within convenience stores for the concentrated display of certified healthy foods [5]. By 2024, the initiative had been expanded and operationally supported across major convenience store chains nationwide [6]. Comparable regulatory approaches to the retail food environment have been adopted elsewhere, though they differ in mechanism rather than constituting direct analogues of the HFC. The United Kingdom restricts the promotion of products high in fat, sugar or salt at prominent in-store locations, which is the closest spatial parallel [7], while measures in the United States operate mainly through product eligibility and labeling rather than store layout [8,9,10]. Retailer-led healthy corner store and convenience store initiatives have also been evaluated in the United States and the United Kingdom [11,12].

Despite these efforts, evidence regarding the effectiveness of Healthy Food Corners in promoting healthy food choice remains limited. Existing initiatives have often focused on the physical placement and categorization of products, while relatively little attention has been paid to the psychological processes through which consumers translate environmental exposure into food choice decisions. In particular, it remains unclear through what cognitive processes spatial and visual elements within the HFC are associated with consumers’ healthy food choice intentions [13]. Moreover, little is known about how consumers evaluate the personal relevance and necessity of healthy food options when exposed to spatially organized healthy food environments. Prior work with Korean convenience store users found that awareness of the need for low-sodium options was a significant predictor of purchase intention [14], suggesting that perceived necessity may be a pivotal but under-examined step in this process. Addressing this gap is essential for understanding how food environment interventions can effectively support healthier dietary decisions in real-world retail settings.

To address this gap, the present study draws on the Stimulus-Organism-Response (S-O-R) framework [15] as its overarching theoretical lens, situating the HFC and its spatial and visual configuration as the environmental stimulus, which is theorized to relate to behavioral intention through consumers’ internal cognitive states. Because a survey cannot manipulate the physical environment, the present study operationalises the stimulus through consumers’ cognitive registration of it, using Awareness of the HFC as an empirical proxy for stimulus exposure rather than as the stimulus itself. Within this framework, we focus particularly on Nudge Theory [16], with visual merchandising (VM) serving as the practical mechanism through which the spatial stimulus is structured and delivered, and on Perceived Necessity as the key cognitive mediator—the point at which consumers evaluate a healthy food choice as genuinely relevant to their own lives.

Understanding how spatial nudges may relate to this evaluative process is important not only for retail strategy but for public health more broadly. Food choices made in convenience stores are often rapid, habitual, and low-deliberation—precisely the conditions under which environmental cues are most influential [17]. If spatial design can be shown to shift these everyday decisions toward healthier options, the cumulative effect on population dietary patterns—particularly among adolescents who rely heavily on convenience stores as food sources—could be substantial [18].

Therefore, the objectives of this study are: (1) to analyze consumers’ awareness and perceived necessity of the Healthy Food Corner; (2) to examine, on an exploratory basis, the associations among HFC awareness, perceived necessity and self-reported purchase intention; (3) to describe consumers’ stated preferences for the spatial and visual features of the HFC, analyzed separately from the association model; and (4) to identify age-related differences in these preferences, with a view to generating hypotheses for targeted food environment design strategies.

## 2. The Stimulus–Organism–Response (S-O-R) Framework in Food Retail Environments

The present study adopts the Stimulus-Organism-Response (S-O-R) framework as its overarching theoretical foundation [15]. Originally developed in environmental psychology, the S-O-R model holds that external environmental stimuli (S) do not directly produce behavioral responses (R); rather, they first activate internal psychological states within the organism (O)—encompassing cognitive appraisals, affective responses, and evaluative judgments—which in turn shape behavioral outcomes [19].

This framework has been widely applied in food retail research to explain how in-store environmental factors influence consumer decision-making [20]. Crucially, the S-O-R model moves beyond the simpler Stimulus-Response (S-R) perspective, which treats consumers as passive agents who react automatically to environmental cues [21]. Instead, the S-O-R model emphasizes the mediating role of internal states, recognizing that the same environmental stimulus may yield different behavioral outcomes depending on how it is cognitively evaluated by the individual [15].

In the context of this study, the three components of the S-O-R framework are operationalized as follows. The *Stimulus* is the Healthy Food Corner itself—its spatial configuration, visual demarcation, labeling, and product display. The *Organism* is Perceived Necessity: the cognitive evaluation through which a consumer judges whether the healthy food option is personally relevant and warranted within their current life context. The *Response* is Purchase Intention—the behavioral outcome that follows from this evaluative process. This mapping provides the structural backbone of the psychological pathway examined in the present study: Awareness → Perceived Necessity → Purchase Intention.

### 2.1. Spatial Nudge in Food Retail Environments

Within the S-O-R framework, Nudge Theory explains *how* the spatial stimulus is designed to activate the organism’s cognitive response. Thaler and Sunstein [16] defined a nudge as any aspect of the choice architecture that alters people’s behavior in a predictable way without forbidding any options or significantly changing their economic incentives [16]. Food choice is a domain particularly well suited to nudge-based intervention: decisions are often made rapidly, automatically, and with limited deliberation, especially in high-traffic retail environments such as convenience stores [13,22].

Unlike coercive policy tools, nudges work by reconfiguring the structural elements of the choice environment—defaults, spatial arrangement, information salience—to gently channel attention toward healthier options without restricting individual freedom [16]. In particular, convenience stores are environments characterized by short dwell times and frequent unplanned purchases, where the physical positioning of products—proximity, order, and visual prominence—exerts a disproportionate influence on selection [17].

The present study conceptualizes the HFC as a *spatial nudge*: a form of choice architecture in which the deliberate demarcation and visual emphasis of a designated zone, rather than individual product placement or labeling alone, functions as the primary nudge mechanism. While previous research has examined isolated nudge elements—shelf position, labels, signage—there is limited empirical work on how the retail space as a whole, when configured as an integrated “choice environment,” relates to consumer cognition [23]. This integrated spatial framing is a key contribution of the present study.

Spatial nudges are frequently implemented through visual merchandising (VM) strategies. Visual merchandising refers to the deliberate design and organization of products and store environments to attract attention and influence consumer responses [24]. Within food retail environments, VM elements such as product visibility, shelf positioning, zoning, signage, and color cues serve as tangible mechanisms through which spatial nudges are communicated and experienced. Prior research has shown that visual attention directed by in-store displays and spatial organization plays a critical role in linking environmental exposure to consumer behavior [25,26].

For health-related foods, visual emphasis through dedicated zones, strategic shelf placement, prominent signage, and color cues can increase product salience, activate health-related associations, and encourage consumers to evaluate healthier options as intentional and meaningful choices rather than incidental purchases [27,28].

Taken together, the Healthy Food Corner may be understood as an integrated spatial nudge intervention implemented through visual merchandising elements that shape how consumers perceive and evaluate healthy food options within the retail environment. However, environmental exposure alone does not necessarily translate into behavioral change. Consumers must first interpret and evaluate the relevance of the available healthy food options, suggesting the importance of an internal cognitive process through which environmental cues are transformed into behavioral intentions.

### 2.2. Perceived Necessity: The Cognitive Mediator

A central premise of this study is that spatial nudges do not influence behavior solely through automatic responses to environmental cues, but through a process of cognitive evaluation. Drawing on the S-O-R model, we position Perceived Necessity as the *organism* variable—the internal evaluative state that mediates between environmental awareness and behavioral intention.

Perceived Necessity, as used in this study, refers to the degree to which a consumer judges a particular food choice to be personally warranted—that is, relevant, appropriate, and aligned with their current life context and health goals. This construct is conceptually distinct from related variables in the consumer behavior literature. It differs from *Perceived Value* [29], which emphasizes a cost–benefit trade-off; from *Attitude* [30], which reflects a stable evaluative disposition; and from *Perceived Healthiness* [28], which concerns beliefs about a product’s nutritional quality. Rather, Perceived Necessity captures an in-context, situational judgment: not “Is this food good for me in general?” but “Do I need this, right now, for myself?”

This evaluative step may therefore be an important condition for a nudge to be associated with behavior, although the present cross-sectional design cannot test that proposition. Research on healthy eating goals suggests that nudge effectiveness is conditional on alignment between environmental cues and consumers’ internalized health goals—without this alignment, cues may fail to promote healthier choices—and that behavioral intentions are most robustly activated when a nudge connects an available option to an individual’s personal health goals, a process that aligns with what we term Perceived Necessity [31].

### 2.3. Purchase Intention and Healthy Food Choice

Purchase intention is widely recognized as one of the strongest predictors of future consumer behavior and has been extensively used in food choice and health behavior research. It reflects an individual’s subjective likelihood or willingness to purchase a particular product and serves as an important indicator of behavioral outcomes when actual purchasing behavior cannot be directly observed [30]. Previous studies have shown that purchase intention is positively associated with subsequent food selection and consumption behaviors, particularly in retail environments where purchasing decisions are made rapidly and repeatedly.

Within the S-O-R framework, purchase intention represents the behavioral response (R) that follows consumers’ cognitive evaluations of environmental stimuli. In the context of the present study, purchase intention reflects consumers’ willingness to purchase foods displayed within the Healthy Food Corner. As the Healthy Food Corner is designed in ways that may be associated with healthier food-choice intentions through spatial and visual cues, examining purchase intention provides an indicator of consumers’ self-reported behavioral response to the HFC. Accordingly, purchase intention is conceptualized as the final outcome of the psychological pathway linking awareness of the Healthy Food Corner and perceived necessity.

Accordingly, this study proposes and empirically examines the following sequential pathway:

**Awareness of the HFC → Perceived Necessity (O) → Purchase Intention (R)**

This pathway situates the HFC within the S-O-R framework, with the caveat that Awareness indexes consumers’ cognitive registration of the stimulus rather than the physical stimulus itself, and provides an empirically tractable model for examining how spatial food environment interventions may relate to healthier food choices at the point of purchase.

Based on the S-O-R framework and the conceptualization of the spatial nudge, this study proposes the following hypotheses:

**Hypothesis 1** **(H1).** *Consumer awareness of the Healthy Food Corner will be positively associated with their perceived necessity of healthy food choices (Organism).*

**Hypothesis 2** **(H2).** *Perceived necessity (Organism) will be positively associated with purchase intentions toward healthy foods (Response).*

**Hypothesis 3** **(H3).** *Awareness is expected to be associated with purchase intention indirectly through perceived necessity.*

## 3. Methods

### 3.1. Study Design

This study employed a cross-sectional online survey design to examine the associations among awareness of the Healthy Food Corner (HFC), perceived necessity and self-reported healthy food purchase intention, and, separately, consumers’ stated preferences for the spatial and visual features of the HFC. Drawing on the S-O-R framework, the HFC and its spatial and visual configuration are conceptualized as the environmental stimulus, while self-reported Awareness is treated as an empirical proxy for consumers’ cognitive registration of prior exposure. Perceived Necessity represents the organism-level evaluative state, and Purchase Intention represents the response. The study focuses on examining associations among self-reported constructs rather than testing the effects of environmental cues or comparing pre- and post-intervention outcomes.

### 3.2. Participants

Participants were 409 consumers aged 14 to 59 years residing in the Seoul metropolitan area (Seoul, Gyeonggi, and Incheon), all of whom had visited a convenience store at least three times in the preceding three months. Enrollment in the online research panel was restricted to individuals aged 14 years and older, in line with the minimum age for independent online panel registration in Korea. Data were collected via an online survey administered by a professional research agency (DataSprings Inc., Seoul, Republic of Korea) between 25 September and 5 October 2023. The sample comprised 200 adolescents (48.9%) and 209 adults (51.1%), with a balanced gender distribution (204 males, 49.9%; 205 females, 50.1%). Informed consent was obtained from all participants before any data were collected. On the survey platform, adults and participants under 18 years of age were directed to separate entry links. Adult participants were presented with an information sheet describing the purpose and background of the study, the voluntary nature of participation and the right to withdraw, and could proceed to the questionnaire only after ticking a box indicating consent. Participants under 18 years of age, and their legal guardians, were provided with the same information in advance; on the minor-specific link the information sheet was followed by an electronic signature field in which both the participant and the participant’s legal guardian had to enter a signature together with their name and the date before the questionnaire could be displayed. Participation therefore required the assent of the minor in addition to the consent of the legal guardian. Respondents were informed in advance that they could withdraw at any point by closing the survey window, in which case no data would be retained, and that responses could not be withdrawn once the questionnaire had been submitted. The study protocol, including these consent procedures, was reviewed and approved by the Institutional Review Board of Ewha Womans University (IRB No. ewha-202309-0035-01). Sample size was determined a priori using G*Power 3.1.9.7 for a one-way fixed-effects ANOVA (effect size f = 0.15, α = 0.05, power = 0.85), indicating a minimum of 402 participants, with the recruitment target inflated to allow for anticipated attrition. An age quota was applied: 200 adolescent respondents were specified in advance because weekly convenience-store visit frequency is highest in this age group, and this quota was met exactly, while adult recruitment yielded 209. Prior experience with a Healthy Food Corner was not an eligibility requirement, which is why most respondents reported none. All 409 returned records were complete on the analysis variables and none were excluded after data collection.

### 3.3. Measures

All items were measured on a 7-point Likert scale (1 = strongly disagree, 7 = strongly agree). Scale items were selected and adapted from validated instruments in prior research [18,32,33,34], with adaptations made to reflect the HFC context. Instrument development was additionally informed by a preceding qualitative study conducted by the same research team, in which convenience store operators and consumers with experience of the Healthy Food Corner took part in interviews addressing awareness, satisfaction, and perceived needs; the themes arising from those interviews guided the selection and wording of the items concerning HFC awareness, necessity, and design preferences. The instrument was administered in Korean, and items adapted from English-language sources were translated into Korean during instrument development; no formal back-translation procedure was carried out. Neither pilot testing nor cognitive interviewing was conducted on the final instrument. Both are acknowledged as limitations, and future studies should incorporate structured cognitive testing and formal translation procedures to establish item comprehension and response-process validity. The questionnaire comprised seven blocks administered in fixed order: an eligibility screen on convenience-store visit frequency; demographic characteristics; convenience-store usage patterns; food purchasing behavior, including shelf-height preferences presented with in-store photographs; HFC awareness and perceived necessity; evaluation of HFC visual materials; and HFC display-method preferences. The awareness block opened with the heading “What is a Healthy Food Corner?”, a photograph of two operating MFDS Healthy Food Corners in CU and GS25 stores showing their branded overhead signage and shelf strips, and the definition “a corner in which foods that can support the development of sound dietary habits in children are separately displayed and labelled for sale in convenience stores.” The third awareness item additionally listed the qualifying product categories. The visual-materials block presented the actual 2023 HFC strip bands, wobblers and show cards as images, and the display-method block presented each alternative as a paired photograph. Because the definition and photograph preceded the awareness items, the awareness measure is a retrospective self-report of prior familiarity elicited after exposure to the stimulus rather than an unprompted measure of pre-existing awareness. The stimulus materials and full item wording are provided as Supplementary Material.

*Awareness* was assessed using three items addressing overall recognition of the HFC, awareness of the product types displayed, and awareness of the display criteria (e.g., “I am aware of the Healthy Food Corner in convenience stores”). Internal consistency was high (Cronbach’s α = 0.952), and a composite mean score was used in subsequent analyses.

*Perceived Necessity* was measured with a single item assessing the degree to which respondents perceived the HFC as personally necessary (E5).

*Purchase Intention* was measured with a single item assessing the respondent’s intention to purchase products from the HFC (E6).

Although multi-item scales are generally preferred for construct measurement, single-item measures are well established for certain constructs, and the two measures used here differ in how strongly that evidence applies. For purchase intention, single-item operationalization is widely accepted in consumer research. This case meets the “doubly concrete” condition specified by Bergkvist and Rossiter [35]: both the object of judgment (products displayed in the Healthy Food Corner) and the rated attribute (intention to purchase) are concrete and singular rather than abstract or multidimensional. Under precisely these conditions, single-item and multi-item measures have been shown to achieve comparable predictive validity, so a single item entails little loss of information [35,36]. Perceived Necessity was likewise measured with a single item capturing a narrowly defined, in-context evaluative judgment of the personal relevance of the Healthy Food Corner. We note, however, that a single-item mediator does not permit the modeling of measurement error within the indirect pathway; accordingly, the mediation analysis is treated as exploratory (Section 4.4), and the study’s primary contributions concern the descriptive contrast between awareness and perceived necessity, and consumer preferences for HFC spatial and visual design. A further consequence of the single-item operationalisation is that the discriminant validity of Perceived Necessity cannot be established in this study. Because only one indicator was available, it is not possible to demonstrate empirically that Perceived Necessity is distinct from conceptually adjacent constructs such as attitude, perceived value, and perceived healthiness, and the construct is therefore presented here as a theoretically proposed mediator rather than a validated one. Establishing discriminant validity will require development of a multi-item scale and formal testing against these related constructs.

### 3.4. Statistical Analyses

Data were analyzed using SPSS Statistics version 29.0. Descriptive statistics, independent samples *t*-tests, chi-square tests, and Pearson’s correlation analyses were conducted to characterize the sample and examine bivariate relationships among key variables. Effect sizes are reported alongside all inferential tests: Cohen’s d with 95% confidence intervals for independent-samples *t*-tests and Cramér’s V for chi-square tests. Expected cell frequencies were inspected for every chi-square analysis, and where any expected count fell below five a Monte Carlo exact test with 20,000 replicates is reported alongside the asymptotic statistic. The age- and sex-stratified comparisons were exploratory rather than pre-specified and were not adjusted for multiplicity; they are interpreted as hypothesis-generating throughout. Regression assumptions were evaluated using the Breusch–Pagan test for homoscedasticity, the Shapiro–Wilk test for residual normality, Cook’s distance for influential observations, variance inflation factors for multicollinearity, and the Durbin–Watson statistic for independence of residuals. In the final model, homoscedasticity was supported (LM = 4.05, *p* = 0.400), no observation was influential (maximum Cook’s D = 0.14), multicollinearity was negligible (all VIF ≤ 1.24) and residuals were independent (Durbin–Watson = 1.99); residuals departed modestly from normality (W = 0.966, *p* < 0.001; skewness −0.56), which is addressed by the bootstrap inference used for the indirect effect. Sex was coded 0 = male and 1 = female, and age group 0 = minor and 1 = adult, in all regression and PROCESS models. The age-moderated extension was estimated with PROCESS Model 7, in which age group moderates the path from Awareness to Perceived Necessity.

To examine the mediating role of Perceived Necessity in the relationship between Awareness and Purchase Intention, hierarchical multiple regression analysis was first conducted with gender and age group entered as covariates in Step 1, Awareness added in Step 2, and Perceived Necessity added in Step 3.

To directly test the statistical significance of the indirect effect (Awareness → Perceived Necessity → Purchase Intention), bootstrapping-based mediation analysis was subsequently conducted using Hayes’ [37] PROCESS macro (Model 4) with 5000 bootstrap samples and bias-corrected 95% confidence intervals (CIs) [37]. An indirect effect is considered statistically significant when the 95% CI does not include zero. As an exploratory extension, whether the indirect pathway varied by age group (minor vs. adult) was examined with a moderated-mediation model, evaluating the index of moderated mediation and the conditional indirect effects within each age group (5000 bootstrap samples).

To assess the potential for common method bias (CMB), an unrotated exploratory factor analysis was conducted with all study variables, and the number of factors with eigenvalues greater than 1.0 and the variance explained by the first unrotated factor were examined as indicators of potential common method bias. Because Harman’s single factor test is a relatively insensitive diagnostic, the robustness of the findings to CMB was further interpreted in light of the marker-variable logic [38], under which method bias would be expected to inflate correlations uniformly across theoretically unrelated constructs [39].

## 4. Results

The results are presented in five parts. We begin with preliminary analyses addressing common method bias, followed by descriptive statistics and correlations that document the descriptive contrast between awareness and perceived necessity. We then report consumer preferences for the specific spatial and visual elements of the HFC. Finally, as an exploratory step, we present hierarchical regression and bootstrapping analyses probing the psychological pathway (Awareness → Perceived Necessity → Purchase Intention).

### 4.1. Preliminary Analyses

Prior to the main analysis, Harman’s single factor test was conducted to evaluate the potential for common method bias (CMB) [39]. The analysis did not yield a single dominant factor; instead, two components with eigenvalues greater than 1.0 were extracted (3.01 and 1.42), indicating that the data did not exhibit the single-factor structure typically associated with substantial CMB. The first factor accounted for 60.18% of the variance, a value largely attributable to the three Awareness items (E1–E3), which were intentionally designed to measure a single construct and demonstrated excellent internal consistency (Cronbach’s α = 0.952).

As a further sensitivity check, following the logic proposed by Lindell and Whitney [38], the smallest observed inter-construct correlation (r_m_ = 0.19) was used as a conservative proxy for common method variance. Using this conservative proxy, the two theoretically central associations remained statistically significant (Perceived Necessity–Purchase Intention: r = 0.681; r_aej_ = 0.61, *p* < 0.001; Awareness–Perceived Necessity: r = 0.316; r_aej_ = 0.16, *p* = 0.001), whereas the already weak association between Awareness and Purchase Intention became non-significant, a pattern consistent with the exploratory indirect association reported below. Taken together, these findings provide some basis for caution against dismissing the results as entirely artifact-driven. However, the 60.18% of variance accounted for by the first Harman factor remains a meaningful concern rather than reassuring evidence against common-method bias, particularly given the strong necessity–intention correlation (r = 0.681). Common-method variance should therefore be treated as a residual, unresolved limitation inherent to the same-source, same-time self-report design. Future studies should employ procedural remedies such as temporal separation of measurement occasions or multi-source designs to address this limitation more definitively.

### 4.2. Descriptive Statistics, Awareness, and Perceived Necessity

Table 1 summarizes the demographic characteristics of the study participants. The final sample comprised 409 respondents: 200 minors (48.9%) and 209 adults (51.1%), with balanced gender distribution (204 males, 49.9%; 205 females, 50.1%). The majority of respondents (310 participants, 75.8%) reported having no prior experience with the HFC, with the most common reason being unawareness (76.1%).

Table 2 presents descriptive statistics for Awareness and Perceived Necessity by age and gender. Awareness was low across all three items (three-item composite M = 2.90 ± 1.66; item-level means 2.82–2.99), indicating limited prior exposure to the HFC. Minors exhibited significantly lower Awareness than adults (2.51 ± 1.73 vs. 3.45 ± 1.70, *p* < 0.001), while awareness of product types and display criteria was higher among males than females (product types: t = 2.079, *p* = 0.038; display criteria: t = 2.050, *p* = 0.041). In contrast, Perceived Necessity was considerably higher (M = 4.86 ± 1.25), suggesting that even consumers with limited prior awareness evaluated healthy food options as personally relevant once the concept was presented. Women scored significantly higher on Perceived Necessity than men (5.04 ± 1.13 vs. 4.67 ± 1.33, t = −3.096, *p* = 0.002), consistent with gender differences in health-oriented food motivation reported in prior research [40].

### 4.3. Psychological Pathway: Hierarchical Regression Analysis

Prior to regression, the internal consistency of the three Awareness items was confirmed (Cronbach’s α = 0.952), and a composite mean score was used in subsequent analyses. Pearson’s correlation analysis confirmed significant positive associations: Awareness was correlated with Perceived Necessity (r = 0.316, *p* < 0.001) and Purchase Intention (r = 0.189, *p* < 0.001), and Perceived Necessity showed a strong positive correlation with Purchase Intention (r = 0.681, *p* < 0.001), consistent with the proposed S-O-R pathway.

Table 3 presents the results of the hierarchical regression analysis predicting Purchase Intention. In Model 1, demographic covariates (gender, age group) explained 1.6% of the variance (R^2^ = 0.016). Adding Awareness in Model 2 produced a significant incremental increase (ΔR^2^ = 0.036, *p* < 0.001), with Awareness as a significant positive predictor (β = 0.200). In Model 3, the inclusion of Perceived Necessity substantially improved model fit (ΔR^2^ = 0.413, *p* < 0.001), with Perceived Necessity as a powerful predictor of Purchase Intention (β = 0.691). Critically, once Perceived Necessity was entered, the direct effect of Awareness became non-significant (β = −0.033, *p* = 0.414), a pattern consistent with full mediation that we interpret as exploratory (Section 4.4). Age was entered as a covariate, and an exploratory moderated-mediation analysis (with age group as moderator) indicated that the indirect pathway did not differ between minors and adults: the index of moderated mediation was non-significant (index = −0.01, 95% CI [−0.12, 0.10]), and the conditional indirect effects were virtually identical for minors (0.175) and adults (0.177). Age-related differences therefore emerged at the descriptive and preference level (Section 4.2 and Section 4.5) rather than in the strength of the awareness–necessity–purchase intention pathway. This divergence may reflect the limitations of the binary age operationalization (minor vs. adult), which collapses substantial developmental heterogeneity within each group: the minor group spans approximately 14–17 years and the adult group 18–59 years, combining participants with markedly different shopping autonomy, food literacy levels, health concern profiles, and developmental stages. How developmental stage, food literacy acquisition, and cultural context moderate responses to spatial nudges in convenience stores remains an important question for future research using more granular age measures. To confirm that this null moderation result is not an artefact of dichotomizing age, the mediation was re-estimated substituting exact age as a continuous covariate. The indirect effect was essentially unchanged (0.186, 95% CI [0.125, 0.247]) relative to the dichotomous specification (0.188, 95% CI [0.128, 0.251]). Examining awareness across five finer age bands further indicates that the age gradient is not linear: mean awareness rises from 2.40 among 14–17-year-olds to 3.06 (18–24), 3.52 (25–34) and 3.58 (35–44) before falling to 3.13 among 45–59-year-olds (one-way ANOVA F = 10.75, *p* < 0.001), so awareness increases sharply after adolescence and then plateaus rather than rising steadily with age.

**Table 3:** Results of Multiple Linear Regression Analysis Among Awareness, Perceived Necessity, and Purchase Intention of the Healthy Food Corner.

Variables	Model 1 β (*p*)	Model 2 β (*p*)	Model 3 β (*p*)
*Control Variables*			
Gender	0.106 (*p* = 0.03)	0.127 (*p* = 0.01)	−0.002 (*p* = 0.96)
Age	0.065 (*p* = 0.19)	0.006 (*p* = 0.91)	0.012 (*p* = 0.75)
*Independent Variable*			
Awareness (Mean)	—	0.200 (*p* < 0.001)	−0.033 (*p* = 0.41)
*Mediating Variable*			
Perceived Necessity	—	—	0.691 (*p* < 0.001)
*R* ^2^	0.016	0.052	0.465
Adj. *R*^2^	0.011	0.045	0.459
Δ*R*^2^	0.016	0.036	0.413
*F* change (*p*)	3.199 (*p* = 0.04)	15.423 (*p* < 0.001)	311.763 (*p* < 0.001)

Standardized β coefficients reported with exact *p* values. Two-tailed tests. Path estimates in Table 4 are unstandardized PROCESS coefficients and therefore differ numerically from these standardized weights.

**Table 4:** Bootstrapping Mediation Results: Indirect Effect of Awareness on Purchase Intention via Perceived Necessity (N = 409, Bootstrap = 5000).

Path	β	SE	95% BootLLCI	95% BootULCI	*p*
Path a: Awareness → Perceived Necessity	0.253	0.036	0.181	0.324	<0.001
Path b: Perceived Necessity → Purchase Intention	0.744	0.042	0.661	0.826	<0.001
Path c: Awareness → Purchase Intention (total)	0.161	0.041	0.080	0.242	<0.001
Path c′: Awareness → Purchase Intention (direct)	−0.027	0.033	−0.091	0.037	=0.414
Indirect effect (a × b)	0.188	0.031	0.128	0.251	—

Note. Coefficients are unstandardized PROCESS estimates and therefore differ in scale from the standardized β weights reported in Table 3. A CI not containing zero indicates a statistically significant indirect effect (a pattern consistent with full mediation, interpreted as exploratory). Bootstrap sample = 5000; bias-corrected 95% confidence intervals.

### 4.4. Bootstrapping Mediation Analysis (Exploratory)

Because the mediator was measured with a single item, the following analysis is reported as an exploratory probe of the proposed pathway rather than as a definitive test of mediation. To formally test the significance of the indirect effect, bootstrapping-based mediation analysis was conducted [37] (Model 4; 5000 resamples; bias-corrected 95% CI) [37]. The indirect effect of Awareness on Purchase Intention via Perceived Necessity was statistically significant (indirect effect = 0.188, SE = 0.031, 95% CI [0.128, 0.251]). As the confidence interval does not include zero, the exploratory mediation hypothesis is supported. The direct effect of Awareness after controlling for Perceived Necessity was non-significant (c′ = −0.027), a pattern consistent with full mediation. Interpreted cautiously, these findings are consistent with the S-O-R framework pathway in which awareness of the HFC, serving as a proxy for exposure to the spatial stimulus, is associated with perceived necessity (O), which in turn is associated with purchase intention (R)—though the cross-sectional design and single-item mediator preclude causal conclusions. Given the cross-sectional design, the simultaneous measurement of all variables, and the single-item mediator, this pathway should be regarded as a hypothesis-generating result rather than confirmation of a causal mechanism. Alternative orderings are equally plausible; for instance, consumers already motivated toward healthy eating may simultaneously report greater perceived necessity and stronger purchase intention, independently of HFC awareness.

### 4.5. Consumer Preferences for Spatial and Visual Elements

This section examines consumer preferences for specific spatial and visual features of the HFC, including preferred store locations, structural configurations, visual cues, and shelf placement.

#### 4.5.1. Store Usage Behavior and Preferred Locations

Regarding the store sections most frequently browsed, respondents predominantly visited beverage refrigerators (35.9%) and RTE food refrigerators (32.5%). Consistent with these traffic patterns, the most preferred location for the HFC was the RTE food refrigerator section (36.4%), followed by dairy refrigerators (17.8%) and beverage refrigerators (17.4%). A significant age-related difference was observed (*p* < 0.001), with adults preferring the RTE and beverage sections and minors preferring the RTE and dairy sections. Detailed data are presented in Appendix A.1 (Table A1) and Appendix A.2 (Table A2).

#### 4.5.2. Structural Configuration and Display Orientation

**Figure 1:**
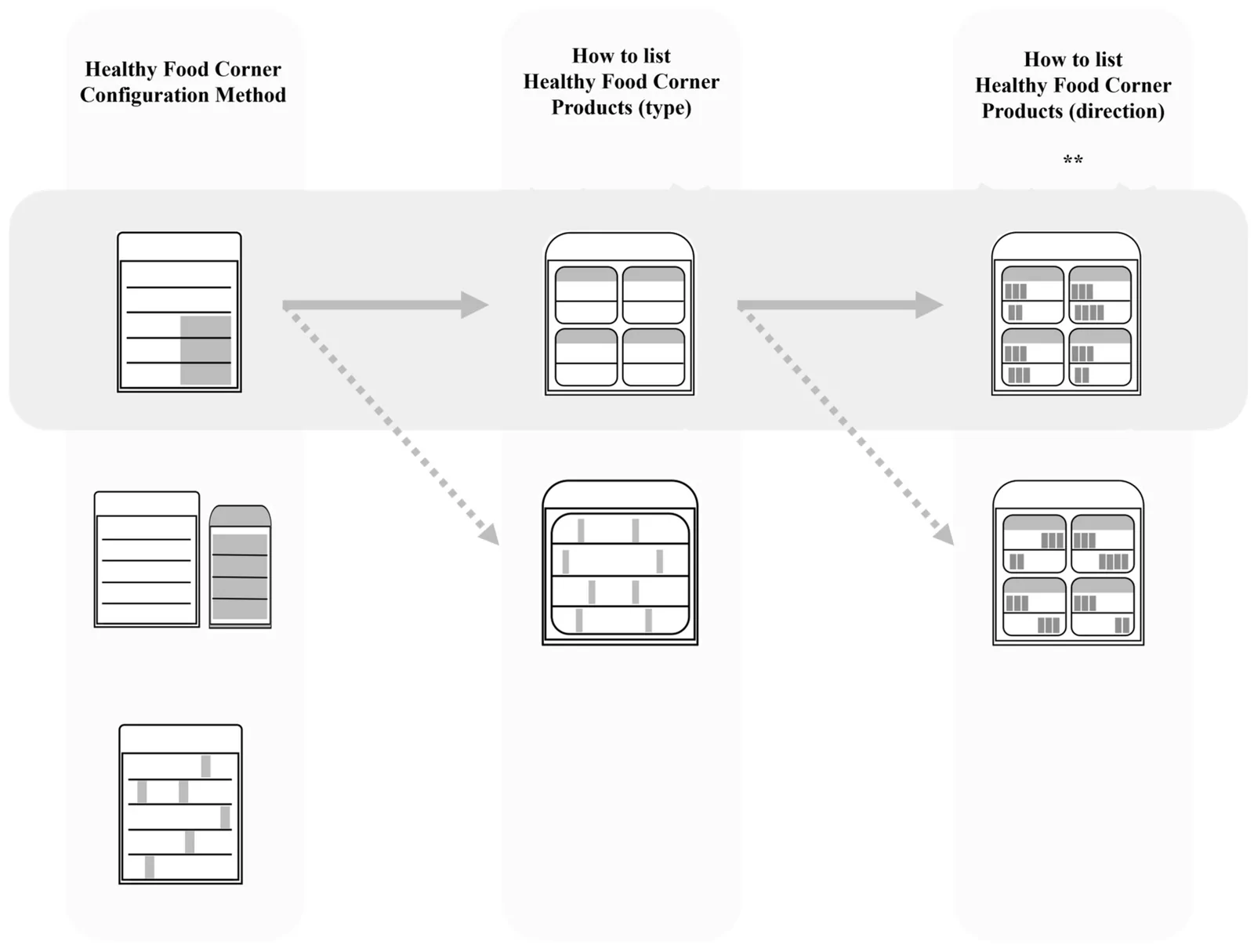
Sequential preferences for the structural configuration of the Healthy Food Corner, including section placement, product grouping, and display orientation; ** *p* < 0.01 for chi-square test of age-group difference in display orientation preference.

Figure 1 depicts the sequential preferences regarding the structural configuration, product grouping, and display orientation of the HFC. Approximately half of the respondents (48.7%) preferred establishing a section within general corners, whereas 40.1% preferred an independent healthy food refrigerator. No significant differences were observed by age or gender. Among respondents who preferred establishing a section within general corners (N = 199), the vast majority (93.5%) favored product grouping by category (e.g., healthy drinks placed alongside regular drinks). This preference was consistent across all groups; notably, among minors, 92.2% of males and 100.0% of females selected this as their first choice.

Regarding display orientation, among those who preferred grouping by type (N = 186), the majority (78.0%) preferred horizontal grouping (left/right). This was the top choice for both minors (68.1%) and adults (88.0%). Within this subgroup, adults were significantly more likely than adolescents to prefer horizontal grouping (88.0% vs. 68.1%; χ^2^ = 10.78, *df* = 1, *p* = 0.001, Cramér’s V = 0.241). We note that the previously reported value of *p* = 0.078 corresponded to the full sample rather than to this subgroup and has been corrected.

#### 4.5.3. Visual Cues: Labeling, Material, and Color

Regarding labeling methods (Table 5), the most preferred option was “zoning labels for healthy food” (74.1%). By age group, 70.0% of minors and 78.0% of adults selected this method, although this difference did not reach statistical significance (χ^2^ = 5.32, *df* = 2, *p* = 0.070, Cramér’s V = 0.114). This suggests that clearly demarcated spatial labeling is preferred by consumers as a signal of the HFC as a distinct, health-promoting zone—a preference consistent with the role of VM as a cognitive scaffold within the S-O-R framework, though its behavioral effectiveness requires experimental verification.

**Figure 2:**
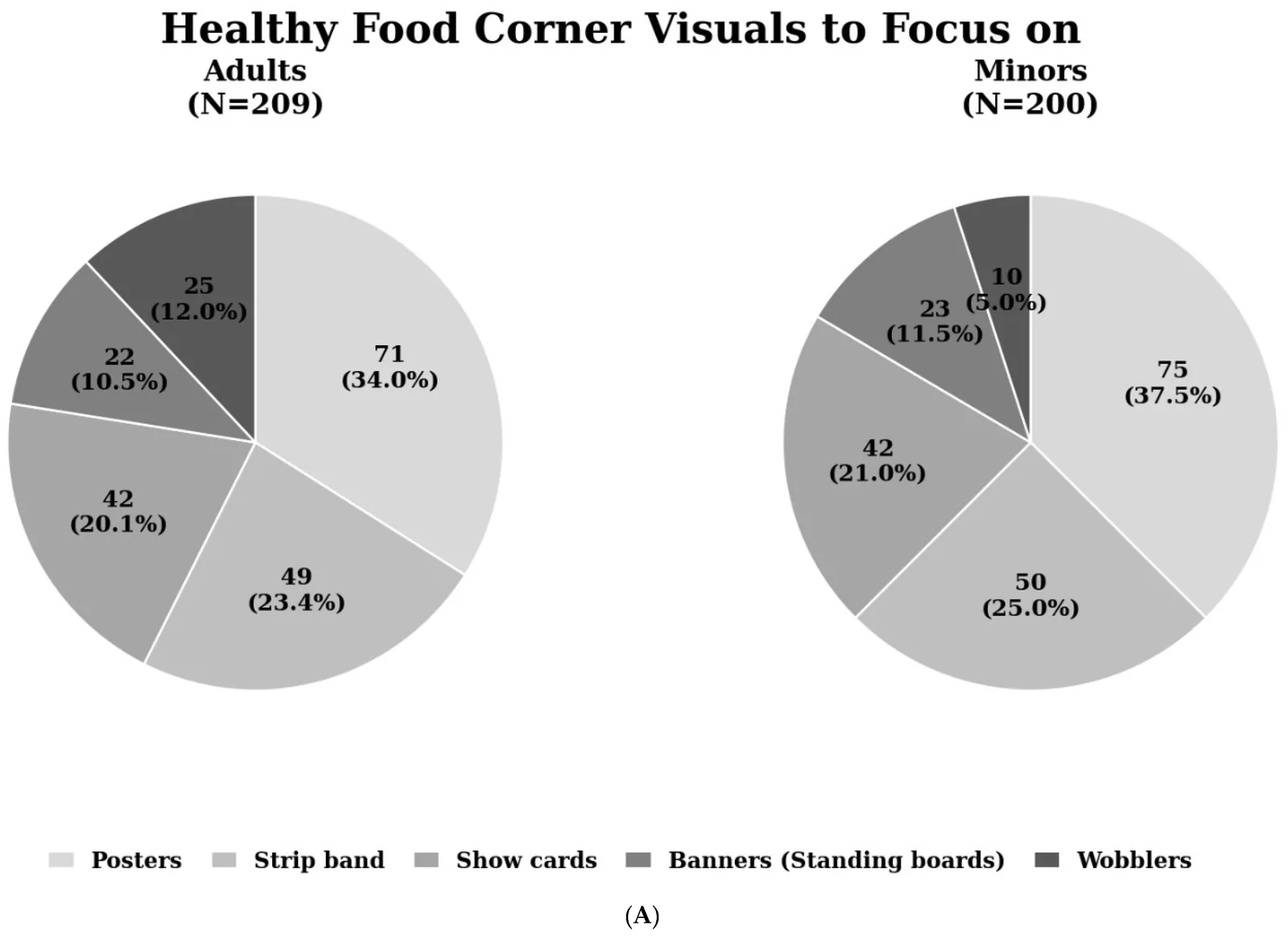

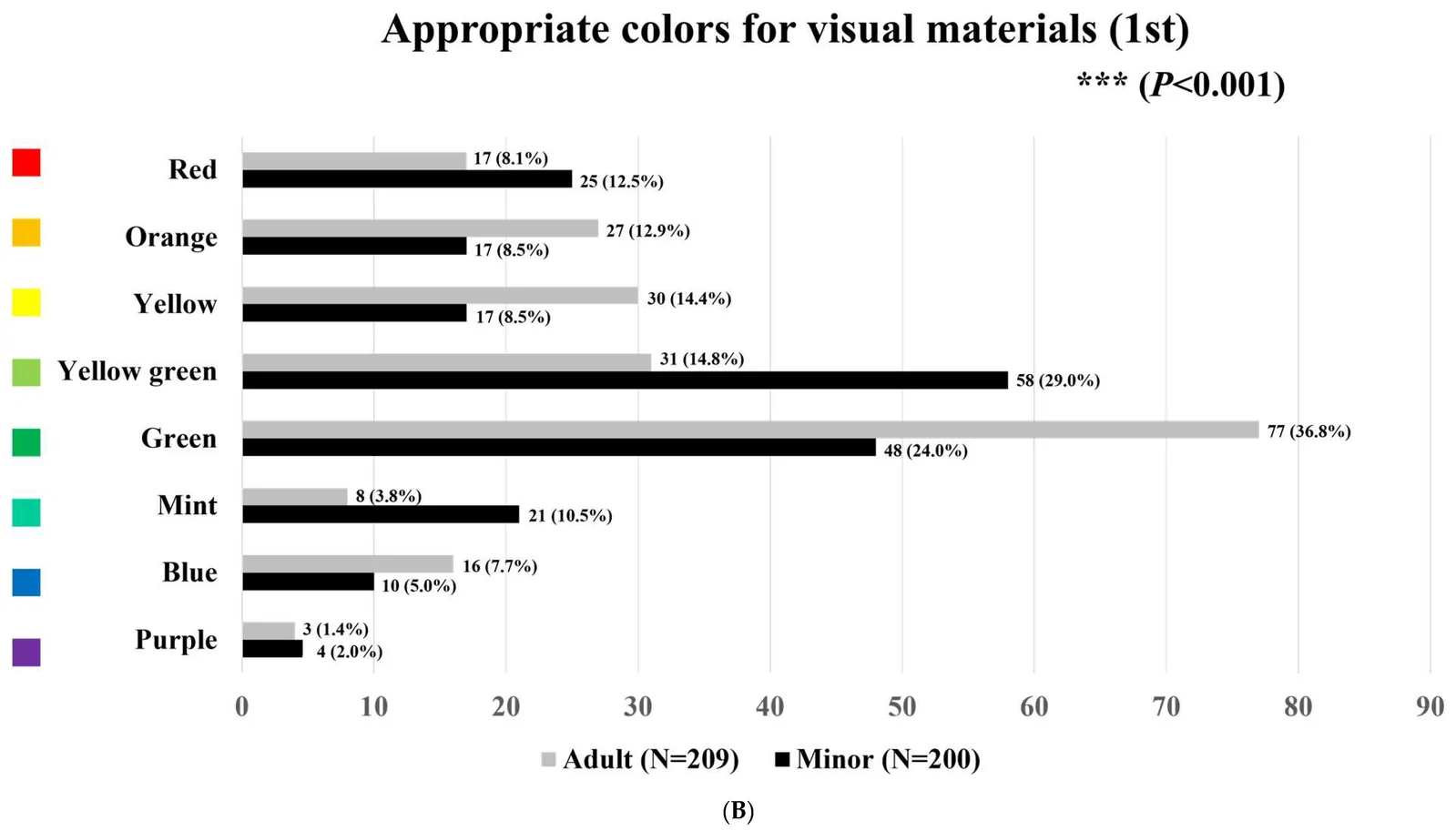
(**A**) Healthy Food corner Visuals to Focus on. (**B**) Appropriate colors for visual materials (1st).

Figure 2 presents preferences for visual cues, specifically visual material types (A) and colors (B). Regarding visual material types (Figure 2A), posters were the most frequently preferred option (35.7%). This indicates that fixed visual media such as posters receive relatively high attention in recognizing the HFC, with similar response patterns observed across age and gender groups.

Regarding color preferences (Figure 2B), the green spectrum was dominant, with Green (30.6%) and Light Green (21.8%) most frequently selected. The predominance of green aligns with prior research demonstrating that green color schemes activate health-related associations and positively influence perceptions of food healthfulness [28]. Minors showed a more balanced preference for Light Green (29.0%) and Green (24.0%), whereas adults showed a distinct preference for standard Green (36.8%), a statistically significant difference (χ^2^ = 29.48, *df* = 7, *p* < 0.001, Cramér’s V = 0.268). Because two cells had expected counts below five, a Monte Carlo exact test was also computed (*p* = 0.018), and the significance of the age difference is unchanged.

#### 4.5.4. Physical Accessibility (Golden Zone)

**Figure 3:**
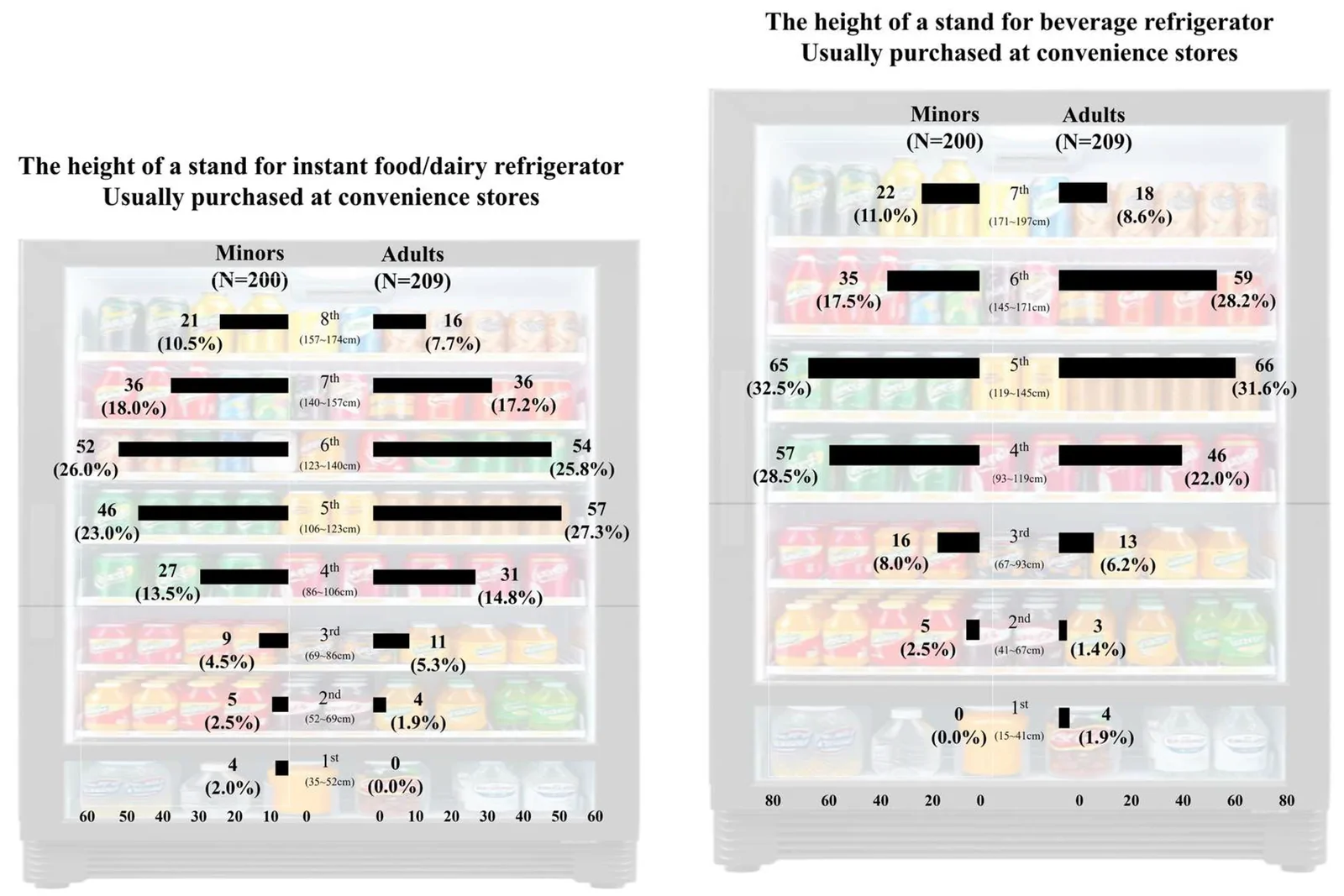
The heights of a stand usually purchased at convenience stores.

Figure 3 depicts preferred shelf heights in refrigerators typically used for purchases. For RTE food and dairy refrigerators, the middle shelf (6th shelf position) was most preferred (25.9%). For beverage refrigerators, the 5th shelf was most preferred. Respondents therefore preferred product placement in the visually accessible mid-level “Golden Zone,” a preference that may inform future HFC design; whether such placement affects attention or purchasing was not tested here. Significant age-related differences in preferred shelf heights were observed across both refrigerator types.

## 5. Discussion

This study examined how consumers perceive the Healthy Food Corner (HFC) in convenience stores as a spatial nudge, and how awareness of it is associated with self-reported purchase intention. Drawing on the Stimulus-Organism-Response (S-O-R) framework [15], we conceptualized the HFC as an environmental stimulus and investigated the associations between HFC awareness, perceived necessity, and consumer purchase intention. The findings revealed an exploratory indirect association of Perceived Necessity in the relationship between Awareness and Purchase Intention, and identified consumer preferences for specific spatial and visual design elements. Together, these results contribute to understanding how consumers perceive and evaluate deliberately designed retail food environments—a question with implications for both individual dietary behavior and population-level public health, although the present design does not test effects on actual food choice. The following sections discuss the theoretical, practical, and policy implications of these findings.

### 5.1. Theoretical Implications

The primary contributions of this study are its documentation of a pronounced descriptive contrast between low awareness and high perceived necessity, and the identification of age-differentiated consumer preferences for HFC spatial and visual design—findings that stand independently of the exploratory mediation analysis. Although awareness of the HFC was low, consumers nonetheless rated it as highly personally necessary, and they expressed clear, age-patterned preferences for how the HFC should be spatially and visually designed. Building on these descriptive findings, and drawing on the S-O-R framework as its theoretical backbone, the study further moves beyond the implicit Stimulus-Response (S-R) assumption—that environmental cues automatically trigger behavioral outcomes—to suggest, on an exploratory basis, that an intervening organism-level cognitive process may be involved [15,21].

Specifically, the exploratory mediation analysis indicated that Awareness was not directly associated with Purchase Intention (Path c′ = −0.027, ns) but was linked to it indirectly through Perceived Necessity (indirect effect = 0.188, 95% CI [0.128, 0.251]). This pattern, consistent with full mediation, is congruent with positioning Perceived Necessity as the organism (O) in the S-O-R chain: the cognitive evaluation through which consumers determine whether a healthy food option is personally warranted before acting. With the caveat that the mediator was single-item and the design cross-sectional, the findings suggest that the observed pattern is consistent with the possibility that spatial nudges in food retail environments may not exert their associations through passive exposure alone, but may involve consumers’ internal evaluative processes [23].

This finding extends the nudge literature in an important direction. Much of the existing nudge research in retail contexts has documented that environmental manipulations—product placement, labeling, default options—can shift behavior, but has given relatively little attention to the psychological mechanisms that explain why and when these effects occur [13]. The present study contributes by suggesting, on an exploratory basis, that the association between self-reported awareness of the HFC and self-reported purchase intention may involve an internal cognitive appraisal of personal necessity rather than awareness alone—though this interpretation must be treated as hypothesis-generating given the cross-sectional design and single-item mediator. The findings are consistent with perspectives on context-dependent, heuristic decision-making under bounded rationality [41,42], but alternative causal orderings cannot be excluded: for example, consumers already motivated toward healthy eating may simultaneously report greater necessity and stronger purchase intention, independent of HFC awareness. Definitively establishing the causal direction of these associations requires longitudinal designs tracking consumers before and after HFC implementation, or experimental studies incorporating randomized exposure conditions and actual sales or purchase outcomes.

Furthermore, this study advances the theoretical treatment of Visual Merchandising (VM) in the context of healthy food promotion. Rather than treating VM as a peripheral aesthetic or commercial tool, the findings position it as a constitutive element of the spatial stimulus—the concrete mechanism through which the nudge is delivered and experienced. Consumer preferences for clear zoning labels, horizontal product arrangement, and health-associated green color schemes identify candidate visual features for future experimental investigation. Whether these features influence cognitive interpretation or purchasing behavior was not tested in the present study. This interpretation aligns with research showing that color and visual organization influence health-related product perceptions and perceived product distinctiveness [27,28].

Finally, the convergence of hierarchical regression and bootstrapping analyses [37] in pointing to the same indirect pathway lends preliminary support to the proposed S-O-R account of how spatial food environment interventions may operate, while the single-item mediator means this account is best regarded as a hypothesis for confirmatory, multi-item testing in future work [37].

### 5.2. Implications for Healthy Food Environment Design

From a practical standpoint, the findings offer a preference-based starting point for designing HFCs, subject to behavioral verification. The present findings suggest that the perceived value of the HFC may not depend solely on its installation, but on whether consumers clearly perceive the space and evaluate it as a personally relevant option. Based on stated preferences, clearly demarcated, visually distinct zones appear to be preferred over dispersing healthy foods across existing shelves; however, whether such demarcation translates into actual behavioral change requires verification through field experiments.

The preference data offer specific design recommendations. The RTE food refrigerator section emerged as the most preferred location for the HFC (36.4%), consistent with traffic patterns showing that this is where consumers most frequently browse and purchase. Placing the HFC at this consumer-preferred high-traffic location may increase the likelihood that spatial cues are encountered during routine shopping; however, neither exposure nor its consequences were measured in the present study. Beyond location, the design of the HFC itself matters: consumers consistently favored clearly demarcated zoning labels (74.1%), horizontal product grouping by category (93.5%), and green color schemes—all of which function as visual signals that distinguish the HFC as a meaningfully different, health-promoting zone.

The Golden Zone findings further underscore the importance of shelf placement as a design variable. Positioning healthy products at eye-level middle shelves—where visual accessibility is highest—can significantly increase the likelihood of spontaneous engagement, particularly in environments characterized by short dwell times and low deliberation [17]. Together, these design elements represent candidate environmental cues rather than merely aesthetic choices; whether they shape the cognitive conditions under which consumers evaluate healthy foods as personally necessary was not tested here and requires experimental confirmation.

Age-related differences in design preferences further emphasize the need for segmentation strategies in spatial design. Minors and adults differed significantly in preferred shelf heights, color preferences, and preferred locations, suggesting that a one-size-fits-all HFC design may underserve key consumer segments. Given that convenience stores serve as primary food access points for adolescents—a group for whom dietary habits formed during this period can have long-term health consequences—age-sensitive spatial design is a promising candidate for intervention development among younger consumers, to be tested rather than assumed [18].

### 5.3. Policy and Societal Implications

The findings of this study carry meaningful implications for public health policy, particularly in the context of food environment interventions aimed at promoting population-level dietary health. While previous health policies have relied primarily on information provision—nutritional labeling, educational campaigns, or fiscal measures—the present results suggest that environment-based spatial interventions may offer a complementary pathway, though whether they constitute a more effective pathway to behavioral change cannot be established from the present self-report data alone. By restructuring the food choice environment itself, spatial nudges may facilitate dietary decision-making at the moment and place where food choices are actually made [13,16], though whether such restructuring shifts behavior requires experimental verification.

Comparing these findings with healthy food retail interventions implemented internationally further contextualizes the present results. In the United Kingdom, regulations restricting the prominent placement of high-fat, sugar, and salt products in key retail locations have demonstrated that spatial and structural approaches to food environment design can be enacted at scale within existing retail systems [7]. Similarly, evidence from healthy corner store and convenience store interventions evaluated in the United States, the United Kingdom and Japan suggests that spatially organized health cues can raise consumer awareness of healthier options, though effects on actual purchasing behavior are typically modest and context-dependent [11,12,43,44]. The present findings are broadly consistent with this international pattern—consumers recognize and value spatially demarcated healthy food zones—while also highlighting features specific to the Korean convenience store context, such as the centrality of ready-to-eat refrigerator sections and the prominence of age-related preferences that may not generalize to Western retail formats.

This approach is particularly well suited to convenience stores, which are characterized by high accessibility, extended hours, and proximity to residential areas—conditions that make them disproportionately influential in shaping the routine food choices of adolescents and young adults. The present findings raise the possibility that the HFC, when thoughtfully designed, could operate not merely as a product display policy but as an everyday public health measure; whether it is associated with consumers’ evolving cognitive frameworks for evaluating healthy foods [3] remains to be established. Repeated exposure to well-designed spatial nudges may, over time, increase familiarity with healthy options—though longitudinal evidence is needed to establish whether such exposure contributes to sustained dietary change. Realizing this potential, however, depends on the retail side as much as on the consumer side. Evidence synthesized across reviews of healthy food retail interventions indicates that retailer knowledge and preferences, profitability, in-store infrastructure, organizational support, and enabling policy all shape whether such initiatives are implemented, sustained, and scaled up [45]. Aligning HFC guidelines with these operational realities—by specifying feasible zoning and signage standards and by supporting store-level execution—would help the HFC evolve from a pilot display into a durable feature of the retail food environment.

The study also highlights the equity dimension of food environment design. The finding that adolescents—who may have less developed capacity for deliberate health-oriented decision-making—respond differentially to spatial cues underscores the importance of designing HFCs with their specific cognitive and behavioral characteristics in mind. Policy efforts that incorporate age-sensitive spatial design principles into HFC guidelines could help ensure that the health-promoting potential of these environments reaches the populations that stand to benefit most [31].

Finally, the results suggest possibilities for extending these spatial principles to digital food environments. The visual structures and organizational logic identified as preferred by consumers in the physical HFC—clear demarcation, health-signalling colors, and prominent placement—may potentially be applicable to online grocery platforms, convenience store applications, and self-service kiosks. This extension is speculative, since no digital environment was examined here, and whether these principles transfer to interfaces that lack the physical and spatial affordances of a store shelf should be examined in future research.

### 5.4. Limitations and Directions for Future Research

Despite its contributions, this study has several limitations that should be acknowledged.

First, the sample was recruited exclusively from the Seoul metropolitan area (Seoul, Gyeonggi, and Incheon), which limits the representativeness of findings even within Korea, where convenience store usage patterns and healthy eating norms may differ across urban and rural regions. More substantially, the generalizability of these findings to other countries is constrained by the distinctive role that convenience stores play in Korean food culture. In Korea and parts of East Asia more broadly, convenience stores function as primary food provisioning outlets serving a wide demographic range; this stands in marked contrast to the more peripheral role of convenience stores in many Western retail systems. The design preferences and awareness–necessity patterns identified in this study should therefore not be assumed to transfer directly to Western retail contexts, and cross-national replication is needed before broader claims can be made. Furthermore, findings regarding adolescents should be interpreted as particularly exploratory, given that this age group’s responses to spatial nudges may vary considerably with developmental stage, food literacy acquisition, age-specific shopping autonomy, and cultural context—factors that the current binary age categorization (minor vs. adult) cannot disentangle. Future research should expand the sample to include non-metropolitan areas, a broader and more granular age range, and cross-national samples. Several further limitations should be noted. Because respondents were shown a definition and photographs of the Healthy Food Corner immediately before rating their awareness, necessity and design preferences, their responses may partly reflect survey-induced exposure rather than pre-existing perceptions; this concern is greatest for the majority of respondents who reported no prior HFC experience. Recruitment through a non-probability online panel introduces potential non-response and coverage bias, and panel members may differ systematically from the general population in digital literacy and survey-taking behavior. The number of panel members invited in order to obtain the 409 completions was not recorded, so a response rate cannot be reported and the extent of any non-response bias cannot be quantified. The age- and sex-stratified comparisons were exploratory and uncorrected for multiplicity, so individual *p* values close to 0.05 should be treated cautiously even where effect sizes are reported. In the color-preference and corner-area analyses some expected cell frequencies fell below five, and although Monte Carlo exact tests were used, these results remain less stable than the other comparisons. Common-method variance cannot be excluded, as discussed above. No validated multi-item instrument exists for Perceived Necessity, and developing one is a priority for future work. Finally, and most fundamentally, stated design preference is not evidence of nudge effectiveness: whether the configurations preferred here would actually attract attention or change purchasing remains untested.

Second, this study relied on consumers’ self-reported perceptions, perceived necessity, and purchase intentions rather than observed behavioral data. While this approach is appropriate for the exploratory goals of the present study, the gap between stated intention and actual food selection remains a well-documented challenge in food behavior research [46]. Future research should employ longitudinal or experimental designs incorporating actual purchase data—such as sales records before and after HFC implementation—to verify behavioral effects.

Third, the cross-sectional nature of the study precludes causal inference. Although the proposed mediation model was theoretically grounded in the S-O-R framework, the observed indirect effects should be interpreted as associative rather than causal pathways. Future longitudinal or experimental studies are needed to establish the directionality of the proposed relationships.

Fourth, this study focused on spatial arrangement and visual elements as the primary stimulus variables, and could not account for commercial factors such as price, product variety, and promotional intensity. These variables are likely to interact with spatial nudge effects in real retail environments, and their inclusion in future models would contribute to a more complete account of HFC effectiveness.

Fifth, Perceived Necessity and Purchase Intention were each operationalized as a single item in this study. Although prior research suggests that single-item measures can be appropriate for concrete, narrowly defined, and conceptually unidimensional constructs [35,36], single-item mediators preclude assessment of measurement error, and the reliability of the mediation estimates therefore cannot be evaluated. The indirect effect (0.188, 95% CI [0.128, 0.251]) should be interpreted as a preliminary, directionally suggestive association that requires replication using validated, multi-item scales. Future research would benefit from developing and validating a multi-item scale for Perceived Necessity to enable more precise assessment of its mediating role in food choice contexts. Relatedly, the discriminant validity of Perceived Necessity against attitude, perceived value and perceived healthiness could not be assessed with a single indicator, so the construct should be treated as provisional until a multi-item scale has been developed and formally validated.

Finally, future research should examine how repeated exposure to well-designed HFCs influences longer-term dietary behavior and health-related outcomes. Research examining the HFC not as a point-in-time nudge but as a sustained environmental intervention, in order to determine whether repeated exposure may gradually reshape consumers’ food-choice frameworks, would provide stronger evidence for its potential as a public health strategy.

### 5.5. Conclusions

This study examined consumer awareness, perceived necessity, and design preferences regarding Healthy Food Corners (HFCs) in Korean convenience stores, and exploratorily tested an associative pathway within the S-O-R framework. Three key findings can be supported by the available data. First, a descriptive contrast was observed between low HFC awareness (three-item composite M = 2.90) and high perceived necessity (M = 4.86), suggesting that consumers value the concept even with limited prior exposure. Second, consumers consistently preferred HFCs positioned in ready-to-eat refrigerator sections, demarcated with zoning labels and green color schemes, organized horizontally, and placed at eye level; age-related differences in these preferences were observed. Third, an exploratory bootstrapping mediation analysis yielded an indirect associative pattern consistent with the S-O-R framework (indirect effect = 0.188, 95% CI [0.128, 0.251]), in which awareness was associated with perceived necessity, which was in turn associated with purchase intention; this pathway should be treated as hypothesis-generating given the cross-sectional design and single-item measures. Experimental and longitudinal research is needed to determine whether preferred design configurations translate into actual behavioral change and whether the observed associations reflect causal mechanisms. These findings offer practical starting points for HFC design and contribute to understanding how consumers relate to spatially organized healthy food environments in convenience store settings.

## Figures and Tables

**Table 1:** General Characteristics of Study Participants (N = 409).

Variables		N	Percentage (%)
Sex (N = 409)	male	204	49.9
	female	205	50.1
Age (N = 409)	minor	200	48.9
	adult	209	51.1
Experience with HFC (N = 409)	Yes	99	24.2
	No	310	75.8
Reasons for No Experience (N = 310)	Unaware/never heard of it	236	76.1
	Limited product variety	25	8.1
	Uncertain about healthiness	33	10.6
	Too expensive	8	2.6
	Other	8	2.6

**Table 2:** Awareness and Perceived Necessity of Healthy Food Corner.

Variables	Total (n = 409)	Age		t (*p*)	Sex		t (*p*)	Cohen’s d [95% CI]	Cohen’s d [95% CI]
		Minor (n = 200)	Adult (n = 209)		Male (n = 204)	Female (n = 205)		Age	Sex
Awareness of Healthy Food Corner	2.99 ± 1.77	2.51 ± 1.73	3.45 ± 1.70	−5.614 (*p* < 0.001)	3.15 ± 1.81	2.83 ± 1.72	1.846 (*p* = 0.07)	0.56 [0.36, 0.75]	0.18 [−0.01, 0.38]
Awareness of product types displayed in the HFC	2.89 ± 1.74	2.37 ± 1.60	3.39 ± 1.73	−6.238 (*p* < 0.001)	3.07 ± 1.80	2.71 ± 1.67	2.079 (*p* = 0.04)	0.62 [0.42, 0.82]	0.21 [0.01, 0.40]
Awareness of display criteria for products in the HFC	2.82 ± 1.72	2.32 ± 1.60	3.30 ± 1.69	−6.031 (*p* < 0.001)	3.00 ± 1.74	2.65 ± 1.67	2.050 (*p* = 0.04)	0.60 [0.40, 0.79]	0.20 [0.01, 0.40]
Necessity of Healthy Food Corner	4.86 ± 1.25	4.74 ± 1.21	4.97 ± 1.27	−1.845 (*p* = 0.07)	4.67 ± 1.33	5.04 ± 1.13	−3.096 (*p* = 0.002)	0.18 [−0.01, 0.38]	0.31 [0.11, 0.50]
Purchase intention	4.81 ± 1.34	4.72 ± 1.36	4.89 ± 1.32	−1.319 (*p* = 0.19)	4.67 ± 1.42	4.95 ± 1.24	−2.156 (*p* = 0.03)	0.13 [−0.06, 0.32]	0.21 [0.02, 0.41]

Mean ± SD. Exact *p* values reported. Two-tailed independent samples *t*-tests. Awareness rows report the three individual items; the three-item composite used in the regression and mediation analyses is M = 2.90 ± 1.66. Cohen’s d is reported with 95% confidence intervals. All age and sex comparisons were exploratory rather than pre-specified and were not corrected for multiplicity; they should be interpreted accordingly.

**Table 5:** Preferences for Labeling Methods of the Healthy Food Corner.

Variables		Total (n = 409)	Minor (n = 200)	Adult (n = 209)	χ^2^ (*p*)	Male (n = 204)	Female (n = 205)	χ^2^ (*p*)
Labeling methods of the HFC	Zoning labels for Healthy Food	303 (74.1)	140 (70.0)	163 (78.0)	5.319 (*p* = 0.07)	153 (75.0)	150 (73.2)	1.386 (*p* = 0.50)
	Promotional tools (strip band, shelf card, wobbler)	70 (17.1)	43 (21.5)	27 (12.9)		31 (15.2)	39 (19.0)	
	Additional labeling on product packaging	36 (8.8)	17 (8.5)	19 (9.1)		20 (9.8)	16 (7.8)	
Total		409 (100)	200 (100)	209 (100)		204 (100)	205 (100)	

Exact *p* values reported for chi-square tests.

## Data Availability

Data will be made available on request.

## Supplementary Materials

The following supporting information can be downloaded at: https://www.mdpi.com/article/10.3390/nu18182971/s1, Figure S1. Photograph shown to all respondents at the start of Block E: operating MFDS Healthy Food Corners in a CU store (left) and a GS25 store (right); Figure S2. Example of Healthy Food Corner visual materials in situ, shown at the start of Block F; Figure S3. Shelf strip band used in the 2023 Healthy Food Corner (item F1); Figure S4. Wobbler and show card used in the 2023 Healthy Food Corner (items F2 and F3); Figure S5. Munsell colour wheel presented for the colour-preference ranking (item F4); Figure S6. Item G2-1, grouping by food type: grouped by type (upper) versus not grouped (lower); Figure S7. Item G2-2, directional alignment: left/right aligned (upper) versus not aligned (lower); Figure S8. Item G3, layout options presented in order: healthy foods mixed into the general corner; a dedicated section within the general corner; and a stand-alone refrigerator for healthy foods; Figure S9. Item G4, labelling options presented in order: strip bands, show cards and wobblers attached per product; zone marking of the healthy food display area; and separate marking on the product package; Table S1. Full item wording; Table S2. Provenance of adapted items.

## Appendix A

### Appendix A.1

**Table A1:** Convenience store food purchasing behavior.

Variables		Total (n = 409)	Age		χ^2^/*p*	Sex		χ^2^/*p*
			Minor (n = 200)	Adult (n = 209)		Male (n = 204)	Female (n = 205)	
The most frequently browsed store sections (1st)	Ready-to-eat (RTE) food refrigerators	133 (32.5)	56 (28.0)	77 (36.8)	32.483 (*p* < 0.001)	66 (32.4)	67 (32.7)	6.820 (*p* = 0.45)
	Dairy refrigerator	28 (6.8)	12 (6.0)	16 (7.7)		14 (6.9)	14 (6.8)	
	Beverage refrigerator	147 (35.9)	71 (35.5)	76 (36.4)		82 (40.2)	65 (31.7)	
	Frozen/frozen food refrigerator	10 (2.4)	1 (0.5)	9 (4.3)		6 (2.9)	4 (2.0)	
	Instant noodle corner	34 (8.3)	29 (14.5)	5 (2.4)		13 (6.4)	21 (10.2)	
	Snack (chips) corner	40 (9.8)	20 (10.0)	20 (9.6)		17 (8.3)	23 (11.2)	
	Candy, chocolate, jelly corner	15 (3.7)	11 (5.5)	4 (1.9)		5 (2.5)	10 (4.9)	
	Checkout area	2 (0.5)	0 (0.0)	2 (1.0)		1 (0.5)	1 (0.5)	
The most frequently purchased store sections (1st)	Ready-to-eat (RTE) food refrigerators	122 (29.8)	51 (25.5)	71 (34.0)	26.592 (*p* < 0.001)	63 (30.9)	59 (28.8)	4.712 (*p* = 0.69)
	Dairy refrigerator	22 (5.4)	8 (4.0)	14 (6.7)		9 (4.4)	13 (6.3)	
	Beverage refrigerator	159 (38.9)	73 (36.5)	86 (41.1)		84 (41.2)	75 (36.6)	
	Frozen/frozen food refrigerator	10 (2.4)	3 (1.5)	7 (3.3)		6 (2.9)	4 (2.0)	
	Instant noodle corner	40 (9.8)	32 (16.0)	8 (3.8)		18 (8.8)	22 (10.7)	
	Snack (chips) corner	37 (9.0)	20 (10.0)	17 (8.1)		15 (7.4)	22 (10.7)	
	Candy, chocolate, jelly corner	18 (4.4)	13 (6.5)	5 (2.4)		8 (3.9)	10 (4.9)	
	Checkout area	1 (0.2)	0 (0.0)	1 (0.5)		1 (0.5)	0 (0.0)	
Total		409 (100)	200 (100)	209 (100)		204 (100)	205 (100)	

Several categories contain small counts, and approximately one fifth of cells had expected frequencies below five; Monte Carlo exact tests with 20,000 replicates were therefore computed and confirm the reported pattern (browsed sections by age, *p* = 0.007; purchased sections by age, *p* = 0.019; both sex comparisons non-significant). Cramér’s V = 0.28 and 0.25 for the age comparisons.

### Appendix A.2

**Table A2:** Most preferred locations for the Healthy Food Corner.

Variables		Total (n = 409)	Age		χ^2^/*p*	Sex		χ^2^/*p*
			Minor (n = 200)	Adult (n = 209)		Male (n = 204)	Female (n = 205)	
Most preferred locations for the Healthy Food Corner	Ready-to-eat (RTE) food refrigerators	149 (36.4)	62 (31.0)	87 (41.6)	23.537 (*p* = 0.001)	64 (31.4)	85 (41.5)	7.013 (*p* = 0.43)
	Dairy refrigerator	73 (17.8)	34 (17.0)	39 (18.7)		34 (16.7)	39 (19.0)	
	Beverage refrigerator	71 (17.4)	28 (14.0)	43 (20.6)		41 (20.1)	30 (14.7)	
	Frozen/frozen food refrigerator	49 (12.0)	29 (14.5)	20 (9.6)		27 (13.2)	22 (10.7)	
	Instant noodle corner	20 (4.9)	16 (8.0)	4 (1.9)		11 (5.4)	9 (4.4)	
	Snack(chips) corner	13 (3.2)	8 (4.0)	5 (2.4)		7 (3.4)	6 (2.9)	
	Candy, chocolate, jelly corner	16 (3.9)	13 (6.5)	3 (1.4)		10 (4.9)	6 (2.9)	
	Checkout area	18 (4.4)	10 (5.0)	8 (3.8)		10 (4.9)	8 (3.9)	
Total		409 (100)	200 (100)	209 (100)		204 (100)	205 (100)	

All expected frequencies in this table exceeded five, so the asymptotic test is appropriate. Cramér’s V = 0.24 for the age comparison and 0.13 for the sex comparison.
