# Supplementary material for: Healthy Food Corners as Spatial Nudges: Consumer Awareness, Perceived Necessity, and Design Preferences in Convenience Stores
Source: Nutrients. 2026 Sep 10;18(18):2971. doi: 10.3390/nu18182971 (PMC13610724; doi:10.3390/nu18182971)
Supplement: Supplementary file 1 [file nutrients-18-02971-s001.zip › nutrients-4482801-supplementary.pdf]

Supplementary Material

Healthy Food Corners as Spatial Nudges: Consumer Awareness, Perceived Necessity, and Design Preferences in Convenience Stores

Overview

This file provides the stimulus materials shown to respondents and the full wording of the survey instrument, in response to reviewer requests for reproducible documentation of what participants saw and how each item was derived.

The survey was administered online in Korean by DataSprings Inc. between 25 September and 5 October 2023. It comprised seven blocks presented in fixed order: (A) eligibility screening; (B) demographic characteristics; (C) convenience-store usage; (D) food purchasing behaviour, including shelf-height preferences presented with in-store photographs; (E) Healthy Food Corner awareness and perceived necessity; (F) evaluation of Healthy Food Corner visual materials; and (G) Healthy Food Corner display-method preferences.

Because the definition and photographs reproduced in Section S2 appeared at the start of Block E, immediately before the three awareness items, the awareness measure is a retrospective self-report of prior familiarity elicited after exposure to the stimulus rather than an unprompted measure of pre-existing awareness. This is noted as a limitation in the main text.

Stimulus shown before the awareness, necessity and purchase-intention items

Block E opened with the heading "What is a Healthy Food Corner?", the photograph below, and the following definition: "a corner in which foods that can support the development of sound dietary habits in children are separately displayed and labelled for sale in convenience stores."

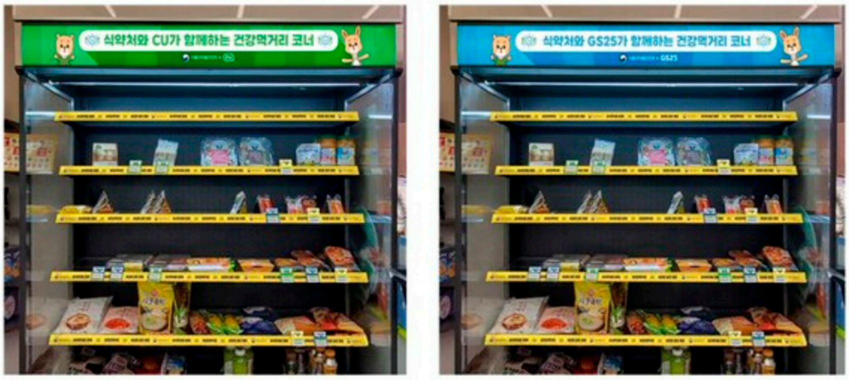

Figure S1. Photograph shown to all respondents at the start of Block E: operating MFDS Healthy Food Corners in a CU store (left) and a GS25 store (right).

The third awareness item additionally listed the qualifying product categories: quality-certified products meeting Ministry of Food and Drug Safety criteria, such as fruit and vegetable juices and fermented milk products; and foods supporting dietary quality, such as fruit excluding sugar-preserved products, salads, and white milk.

Visual materials evaluated in Block F

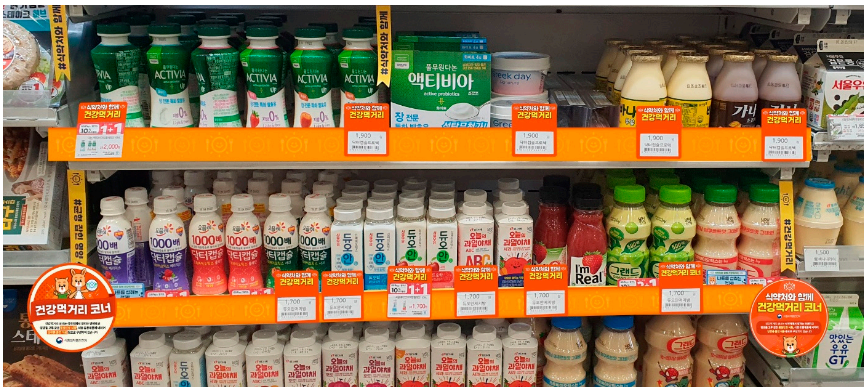

Figure S2. Example of Healthy Food Corner visual materials in situ, shown at the start of Block F.

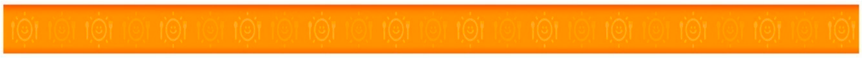

Figure S3. Shelf strip band used in the 2023 Healthy Food Corner (item F1).

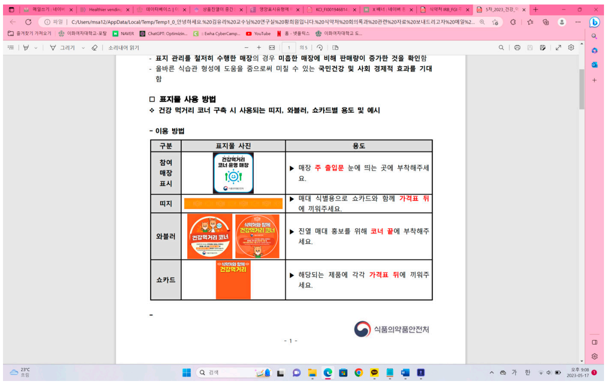

- 표지 관리를 철저히 수행한 매장의 경우 미흡한 매장에 비해 판매량이 증가한 것을 확인함
- 올바른 식습관 형성에 도움을 줌으로써 미칠 수 있는 국민건강 및 사회 경제적 효과를 기대함

□ 표지물 사용 방법

❖ 건강 먹거리 코너 구축 시 사용되는 띠지, 와블러, 쇼카드별 용도 및 예시

- 이용 방법

구분	표지물 사진	용도
참여 매장 표시	건강먹거리 코너 운영 매장	▶ 매장 주 출입문 눈에 띄는 곳에 부착해주세요.
띠지		▶ 매대 식별용으로 쇼카드와 함께 가격표 뒤에 끼워주세요.
와블러	건강먹거리 코너 / 식약처와 함께 건강먹거리 코너	▶ 진열 매대 홍보를 위해 코너 끝에 부착해주세요.
쇼카드	식약처와 함께 건강먹거리	▶ 해당되는 제품에 각각 가격표 뒤에 끼워주세요.

-

식품의약품안전처

- 1 -

Figure S4. Wobbler and show card used in the 2023 Healthy Food Corner (items F2 and F3).

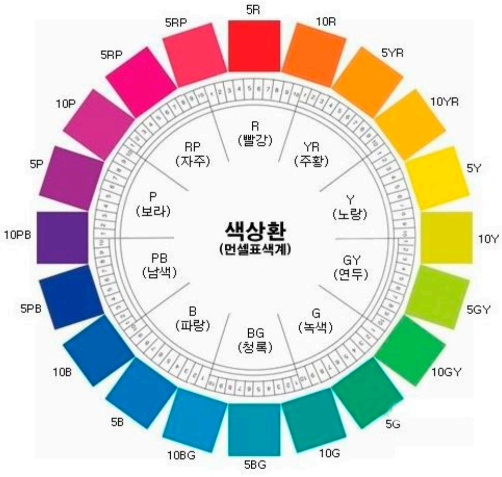

Figure S5. Munsell colour wheel presented for the colour-preference ranking (item F4).

Display alternatives presented in Block G

Each display alternative was presented as a photograph. Respondents chose between paired options for grouping and orientation, and among three options for overall layout and for labelling method.

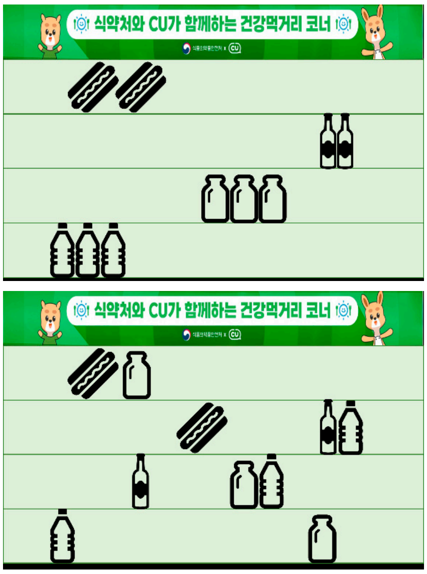

Figure S6. Item G2-1, grouping by food type: grouped by type (upper) versus not grouped (lower).

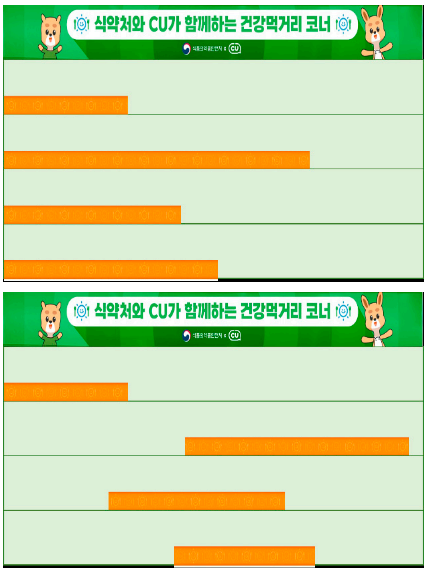

Figure S7. Item G2-2, directional alignment: left/right aligned (upper) versus not aligned (lower).

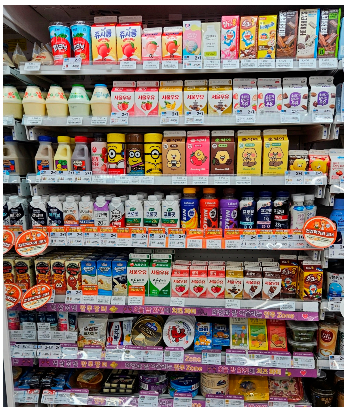

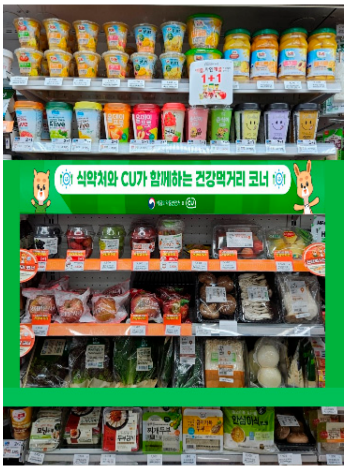

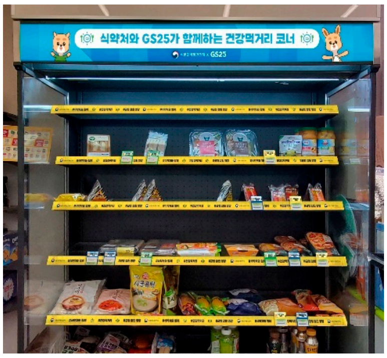

Figure S8. Item G3, layout options presented in order: healthy foods mixed into the general corner; a dedicated section within the general corner; and a stand-alone refrigerator for healthy foods.

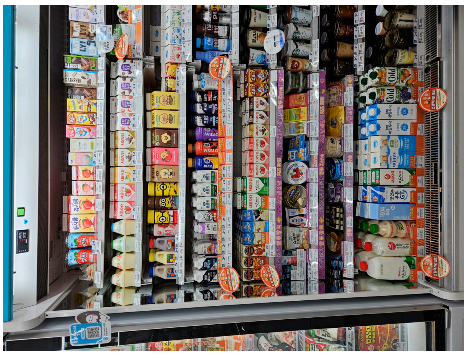

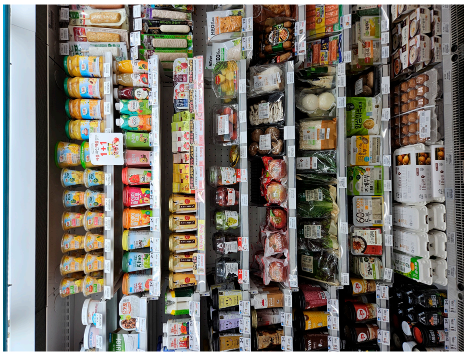

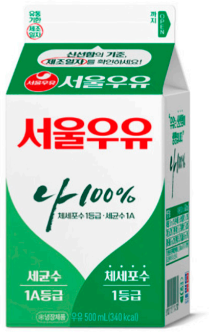

Figure S9. Item G4, labelling options presented in order: strip bands, show cards and wobblers attached per product; zone marking of the healthy food display area; and separate marking on the product package.

Full item wording

All items are listed in the order administered. Block D shelf-height items (D5–D12) were each presented with an in-store photograph of the relevant cabinet, a labelled shelf ladder giving the height range of every shelf position, and a 160 cm human silhouette for scale; the shelf ranges are given in the table.

Table S1 - Full item wording

Block	Item	Korean wording (as fielded)	English translation	Response format
A	A1	최근 3개월간 3회 이상 편의점을 방문하신 경험이 있으십니까?	Have you visited a convenience store at least three times in the past three months?	Yes / No (screen-out)
B	B1	귀하의 성별은 무엇입니까?	What is your sex?	Male / Female
B	B2	귀하의 연령은 어떻게 되십니까? (만 세)	What is your age? (years)	Open numeric
B	B3	귀하의 결혼 여부는 무엇입니까?	What is your marital status?	3 options
B	B4	귀하의 최종학력은 다음 중 어디에 속하십니까?	What is your highest level of education?	5 options
B	B5	현재 귀하의 직업은 무엇입니까?	What is your current occupation?	7 options
B	B6	현재 귀하 세대의 월 평균 소득 수준은 어디에 속하십니까?	What is your household's average monthly income?	6 brackets
B	B7	귀하 가구의 형태는 어디에 속하십니까?	What is your household type?	3 options
B	B8	귀하의 키와 체중을 적어주십시오.	Please report your height and weight.	Open numeric
C	C1	귀하는 편의점을 얼마나 자주 이용하십니까?	How often do you use convenience stores?	5 options
C	C2	귀하가 하루 중 편의점을 가장 많이 이용하는 시간은 언제입니까?	At what time of day do you most often use convenience stores?	5 options
C	C3	귀하가 편의점을 가장 많이 이용하는 요일은 언제입니까?	On which days do you most often use convenience stores?	4 options
C	C4	귀하가 편의점에서 주로 구매하는 식품은 무엇입니까?	Which foods do you mainly purchase at convenience stores?	7 options

C	C5	귀하가 가장 자주 이용하는 편의점은 어디입니까?	Which convenience store chain do you use most often?	5 options
D	D1	귀하는 편의점 방문 시 구매 식품을 미리 결정하십니까?	Do you decide what to buy before visiting a convenience store?	4 options
D	D2	구매를 결정했던 식품 이외의 식품을 구매한 경험이 있다면 어떤 이유로 구매하셨습니까?	If you have bought foods other than those you intended, why?	7 options
D	D3	귀하가 편의점에서 주로 둘러보는 식품 코너를 순서대로 선택해주십시오.	Please rank the store sections you most often browse.	Rank 1st–3rd of 9 sections
D	D4	귀하가 편의점에서 주로 구매하는 식품의 코너를 순서대로 선택해주십시오.	Please rank the store sections from which you most often purchase.	Rank 1st–3rd of 9 sections
D	D5	귀하가 편의점에서 주로 둘러보는 상온 식품 코너 칸의 높이는 어디입니까?	At which shelf height do you most often browse the ambient food section?	9 shelf positions (8–179 cm), with photograph
D	D6	귀하가 편의점에서 주로 구매하는 상온 식품 코너 칸의 높이는 어디입니까?	At which shelf height do you most often purchase from the ambient food section?	9 shelf positions (8–179 cm), with photograph
D	D7	귀하가 편의점에서 주로 둘러보는 즉석식품/유제품 냉장고 칸의 높이는 어디입니까?	At which shelf height do you most often browse the ready-to-eat and dairy refrigerator?	8 shelf positions (35–174 cm), with photograph
D	D8	귀하가 편의점에서 주로 구매하는 즉석식품/유제품 냉장고 칸의 높이는 어디입니까?	At which shelf height do you most often purchase from the ready-to-eat and dairy refrigerator?	8 shelf positions (35–174 cm), with photograph
D	D9	귀하가 편의점에서 주로 둘러보는 음료 냉장고 칸의 높이는 어디입니까?	At which shelf height do you most often browse the beverage refrigerator?	7 shelf positions (15–197 cm), with photograph
D	D10	귀하가 편의점에서 주로 구매하는 음료 냉장고 칸의 높이는 어디입니까?	At which shelf height do you most often purchase from the beverage refrigerator?	7 shelf positions (15–197 cm), with photograph
D	D11	귀하가 편의점에서 주로 둘러보는 냉동식품 냉동고 칸의 높이는 어디입니까?	At which shelf height do you most often browse the frozen food cabinet?	7 shelf positions (38–194 cm), with photograph
D	D12	귀하가 편의점에서 주로 구매하는 냉동식품 냉동고 칸의 높이는 어디입니까?	At which shelf height do you most often purchase from the frozen food cabinet?	7 shelf positions (38–194 cm), with photograph
D	D13	귀하가 편의점에 방문하여 이동하는 순서를 선택해 주십시오.	Please rank the order in which you move through the store.	Rank 1st–8th, with store layout diagram
E	E1	귀하는 편의점의 건강먹거리코너에 대해서 알고 계십니까?	Are you aware of the Healthy Food Corner in convenience stores?	7-point Likert
E	E2	귀하는 편의점의 건강먹거리코너에 진열되는 상품의 종류에 대해서 알고 계십니까?	Are you aware of the types of products displayed in the Healthy Food Corner?	7-point Likert

E	E3	귀하는 편의점의 건강먹거리코너에 상품을 진열하는 기준에 대해서 알고 계십니까?	Are you aware of the criteria by which products are displayed in the Healthy Food Corner?	7-point Likert
E	E4	귀하는 편의점의 건강먹거리코너를 이용한 경험이 있으십니까?	Have you ever used a Healthy Food Corner in a convenience store?	Yes / No
E	E4-2	건강먹거리코너를 이용하지 않은 이유는 무엇입니까?	Why have you not used the Healthy Food Corner?	5 options (asked if E4 = No)
E	E5	귀하는 편의점의 건강먹거리코너가 필요하다고 생각하십니까?	Do you think a Healthy Food Corner is necessary in convenience stores?	7-point Likert
E	E6	귀하는 건강먹거리코너에서 제품을 구매할 의향이 있습니까?	Would you be willing to purchase products from the Healthy Food Corner?	7-point Likert
F	F1	2023년 건강먹거리코너에 부착된 띠지에 만족하십니까?	How satisfied are you with the shelf strip band used in the 2023 Healthy Food Corner?	7-point Likert
F	F2	2023년 건강먹거리코너에 부착된 와블러에 만족하십니까?	How satisfied are you with the wobbler used in the 2023 Healthy Food Corner?	7-point Likert
F	F3	2023년 건강먹거리코너의 제품 앞에 부착된 쇼카드에 만족하십니까?	How satisfied are you with the show card used in the 2023 Healthy Food Corner?	7-point Likert
F	F4	건강먹거리코너의 시각물에 가장 적절하다고 생각하는 색상을 순서대로 선택해주십시오.	Please rank the colours you consider most appropriate for Healthy Food Corner visual materials.	Rank 1st–3rd from colour wheel
G	G1	건강먹거리코너 면적이 어느 정도면 적당하다고 생각하십니까?	What shelf area do you consider appropriate for a Healthy Food Corner?	1–7 shelf units
G	G2-1	식품 유형별 모음 진열: 적절하다고 생각하는 방식은?	Grouping by food type: which arrangement do you consider appropriate?	Grouped by type / Not grouped
G	G2-2	방향 정렬 진열: 적절하다고 생각하는 방식은?	Directional alignment: which arrangement do you consider appropriate?	Left/right aligned / Not aligned
G	G3	다음의 진열 방식에서 어떤 진열 방식을 선호하십니까?	Which of the following layout options do you prefer?	Mixed / Section within general corner / Dedicated refrigerator
G	G4	건강한 먹거리 제품 표시 방법 중 어떤 방법을 선호하십니까?	Which product labelling method do you prefer?	Per-product attachment / Zone marking / Package marking

Provenance of adapted items

The instrument was assembled from previously published measures and adapted to the Healthy Food Corner context. No formal cognitive interviewing was conducted, and this is stated as a limitation in the main text.

Table S2 - Provenance of adapted items

**Construct / item block**	**Source instrument adapted**

Awareness and perceived necessity of the HFC (E1–E5)	Kim & You [32], market segmentation by consumption value among convenience-store PB food consumers; Mumena et al. [33], knowledge, attitudes and practices toward added sugar; Rosi et al. [34], vending-machine availability and consumer awareness
Purchase intention (E6)	Bergkvist & Rossiter [35]; Fuchs & Diamantopoulos [36] — single-item operationalisation for doubly concrete constructs
Convenience-food use and adolescent context (C block)	Park & Lee [18] — Korean adolescents' convenience-food experience
Visual materials and colour (F1–F4)	Sonnenberg et al. [44], traffic-light labelling
